# Rapid Formation and Interfacial Adhesion Enhancement in Zirconium Conversion Coatings on 55AlZnMg-Coated Steel Under a Short H_2_ZrF_6_ Pretreatment

**DOI:** 10.3390/ma19122545

**Published:** 2026-06-12

**Authors:** Xiaonan Zhang, Weixi Zhao, Lin Lu

**Affiliations:** Institute for Advanced Materials and Technology, University of Science and Technology Beijing, Beijing 100083, China; m202411459@xs.ustb.edu.cn (X.Z.); zhaoweixi1220@163.com (W.Z.)

**Keywords:** 55AlZnMg coating, ZrCC, Zirconate, conversion film, dealloying

## Abstract

**Highlights:**

- Short-time pretreatment: a selected H_2_ZrF_6_ bath condition at 40 °C and pH 4 enabled rapid ZrCC surface coverage on 55AlZnMg-coated steel within 60 s.
- Advanced substrate focus: addressed the complex, multiphase microstructure of 55AlZnMg-coated steel, a multiphase Al-Zn-Mg-coated steel substrate where traditional conversion methods typically produce uneven results.
- Substantial adhesion boost: achieved a 64.0% increase in interfacial bond strength between the substrate and an overcoated polyurethane (PU) organic layer.

**Abstract:**

To address the uneven deposition of zirconium conversion coatings on multiphase 55AlZnMg under short pretreatment cycles, this study investigated the time-dependent formation behavior of ZrCC in a selected H_2_ZrF_6_ bath. By precisely controlling the immersion time (20–90 s) and utilizing SEM-EDS and AFM characterization techniques, this study systematically revealed the growth kinetics and film-forming mechanisms of ZrCC on complex alloy surfaces. The results indicate that the Zn-rich phase on the surface of the 55AlZnMg coating, due to its relatively positive potential, preferentially induces the deposition of the film-forming material. Subsequently, dealloying occurs in the Al-rich phase and the Mg/Zn enriched regions, forming Zn-enriched regions that promote the continuous deposition of the film-forming material, ultimately achieving complete surface coverage; the film morphology evolves from an initial needle-like structure to a network structure, eventually forming a nanosheet structure. The film-forming process of ZrCC on the 55AlZnMg substrate surface is primarily driven by selective growth, with electrochemical properties of the alloy phases, significantly enhancing adhesion between the aluminum-zinc-magnesium coating and the overcoat and providing practical guidance for improving surface uniformity and interfacial adhesion of Al-Zn-Mg-coated steel.

## 1. Introduction

To meet the corrosion resistance requirements of metallic materials under various service conditions, various conversion coatings and pretreatment processes have been continuously developed and refined [1,2,3,4,5,6,7,8]. Such coatings typically serve a dual purpose: they act as a physical barrier to block corrosive media and enhance the adhesion of organic coatings through interfacial chemical interactions. Among the many conversion technologies, the chromate conversion process is mature and cost-effective; however, its use has been strictly restricted due to the extreme toxicity of hexavalent chromium and environmental pollution concerns [1,5]. Consequently, zirconate conversion coatings, which combine environmental friendliness with potentially useful interfacial functions, have emerged as an ideal alternative, and the demand for their short pretreatment cycles on diverse metal substrates is growing increasingly urgent. As a highly corrosion-resistant alloy coating, 55AlZnMg has been widely applied in the construction, light industry, home appliance, and automotive sectors [9,10,11,12]. However, compared to traditional hot-dip galvanized steel sheets, the complex microstructure of its surface—characterized by the coexistence of multiple phases—imposes higher demands on the uniformity, adhesion, and surface uniformity and adhesion reliability of the zirconate conversion coating.

Studies have shown that the film-forming kinetics of zirconate conversion coatings are influenced by a combination of factors, including the chemical state of the substrate, conversion time, and the fluid environment. The chemical properties of the substrate are the key determinant of the film-forming process. Cerezo et al. [13,14] found that the interaction between F^−^ and hydroxyl groups on the substrate surface, and its ion-exchange capacity within the oxide lattice are the primary mechanisms. Differences in M–O bond energies (Al–O > Fe–O > Zn–O) among various substrates significantly affect the surface activation rate and film thickness [15,16]. Conversion time is another important parameter that regulates film structure and performance. Andreatta et al. [17] observed during the preparation of Zr/Ti conversion coatings on AA6016 surfaces that, after 120 s of immersion, the coating material primarily deposited on the intermetallic precipitates (IMPs), with a voltaic potential difference of 280 mV; upon extending the treatment to 300 s, the conversion coating completely covered the surface, and the potential difference decreased to 5 mV, indicating that as the treatment time increased, the coating coverage became more complete. Research by Khun et al. [18] on low-carbon steel further demonstrated that surface roughness first increased and then decreased with increasing immersion time, reflecting the evolution of the coating from initial nucleation and cluster growth to a uniform structure [19]. Additionally, agitation can increase the surface F^−^ concentration by enhancing mass transfer, thereby shortening the activation time and extending the effective deposition window for Zr^4+^ [20,21,22,23,24]. However, excessively long conversion times lead to a decline in coating performance. Research by Stromberg et al. [25] indicates that zirconate conversion coatings on galvanized steel surfaces exhibit optimal performance at approximately 8 min; beyond 10 min, localized dissolution results in a porous structure, increased defect density, and a significant decline in protective performance. However, there is currently very little research on zirconate conversion coatings for substrates such as 55AlZnMg, whose electrochemical activity distribution on the coating surface is far more complex than that of traditional substrates.

In addition to the immersion time, acid concentration is also a key factor affecting the film-forming properties of the conversion coating. Acid concentration influences parameters such as ZrF_6_^−^ concentration, Zr^4+^ concentration, F^−^ concentration, and pH [25]. ZrF_6_^−^ in the solution can improve surface wettability [26] and promote surface activation; on the other hand, the presence of Zr-F complexes maintains the solvation of Zr^4+^, prevents premature hydrolysis, and controls the formation of the conversion coating [27]. However, if the concentration of hexafluorozirconic acid (H_2_ZrF_6_) is too high (>10^−2^ M), Zr-F complexation will raise the pH of the precipitate, thereby reducing the Zr content in the conversion coating. When preparing conversion coatings on AM60 surfaces, increasing the H_2_ZrF_6_ concentration in the conversion bath from 0.001 M to 0.1 M resulted in a decrease in the Zr content in the conversion coating from 10 at.% to 1.3 at.% [28]. Furthermore, ZrF_6_ undergoes hydrolysis, a process involving isomeric substitution between F^−^ and OH^−^ [27]. Regarding the role of F^−^ in conversion coating formation, various mechanisms have been proposed in the literature; however, overall, it is detrimental to conversion coating formation and affects the chemical properties and electrochemical behavior of the substrate surface [23,24]. It is worth noting that the aforementioned studies on acid concentration have primarily focused on carbon steel sheets and aluminum-alloy steel sheets, with very few studies conducted on 55AlZnMg-coated steel sheets. Therefore, further investigation is needed to determine whether the acid concentration discussed above is applicable to 55AlZnMg-coated steel sheets.

At the same time, the pH of the conversion bath affects the uniformity and performance of the resulting conversion coating; most studies use a treatment solution with a pH between 3.5 and 4.5. Lunder et al. [29] treated AA6060 with the commercial treatment solution Gardobond X4707^®^ (Chemetall GmbH, Frankfurt am Main, Germany) while adjusting the pH to different levels. The results showed that the coating formed at pH 4 was more uniform and thicker, and exhibited better corrosion resistance. This is because a lower pH may cause the conversion coating to dissolve in the conversion bath, resulting in a coating that cannot adequately protect the substrate. If the pH is too high, the cathodic reaction rate is insufficient to raise the pH at the interface of the zirconium (hydroxide) precipitation, preventing the achievement of localized alkalization conditions [30,31].

Although research on zirconate conversion coatings has made some progress, most results remain confined to laboratory conditions, and the developed process parameters struggle to meet the requirements of industrial-scale continuous production. One of the key issues is the excessively long immersion time; for example, complete film formation can take as long as 300 s [17], far exceeding the typical 1–4 min required in industrial continuous-processing lines [32,33]. Furthermore, while intensification measures such as mechanical agitation can promote film formation, they also increase process complexity and equipment costs, limiting their practical application. Existing research has primarily focused on traditional substrates such as steel plates, aluminum alloys, and ordinary hot-dip galvanized sheets, with a lack of systematic investigation into the unique surface characteristics of 55AlZnMg coatings. The coexistence of Zn-rich, Al-rich, and Mg/Zn-enriched regions in this coating results in a complex distribution of surface electrochemical activity, and the film formation kinetics may differ significantly from those of homogeneous substrates. If existing processes are directly applied, interphase deposition differences can easily lead to issues such as coating non-uniformity and reduced adhesion, ultimately affecting the overall reliability of the protective system.

Therefore, to adapt to short pretreatment cycles of zirconate conversion technology on 55AlZnMg coatings, there is an urgent need to conduct research on film formation mechanisms under conditions relevant to short pretreatment cycles. In this study, zirconium conversion coatings were prepared on 55AlZnMg-coated steel in an H_2_ZrF_6_ pretreatment bath under short immersion times. By systematically varying the immersion time, the time-dependent surface coverage, elemental redistribution, and morphology evolution of the ZrCC layer were investigated. The aim of this work is to clarify the rapid film-forming behavior of ZrCC on the multiphase 55AlZnMg surface and to reveal how phase-dependent electrochemical activity regulates selective deposition and surface homogenization. The possible role of the selected H_2_ZrF_6_ bath chemistry in promoting hydrolysis–precipitation is discussed, while the specific concentration effect requires further verification through concentration-dependent studies.

## 2. Materials and Methods

### 2.1. Materials and Sample Preparation

**Figure 1:**
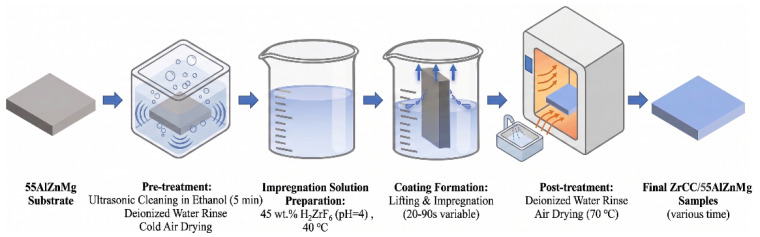
ZrCC/55AlZnMg preparation flow chart.

In this study, 55AlZnMg-coated steel sheets were used as the substrate. The coating composition consisted of 55 wt.% Al, 41.4 wt.% Zn, 2 wt.% Mg, and 1.6 wt.% Si, with an average coating weight of 75 g/m^2^ and a total sheet thickness of 0.6 mm, of which the coating thickness was 30 μm. All samples were ultrasonically cleaned in anhydrous ethanol for 5 min to remove oil and dust from the substrate surface, then rinsed with deionized water and air-dried for later use. Zirconium conversion coatings were prepared by immersing the cleaned 55AlZnMg-coated steel sheets in an aqueous H_2_ZrF_6_ pretreatment bath. The bath was prepared by diluting a commercial 45 wt.% H_2_ZrF_6_ stock solution to a final H_2_ZrF_6_ concentration of 4 wt.%, and the pH was adjusted to 4.0. The treatment was carried out at 40 °C for different immersion times of 20, 30, 40, 60, and 90 s. After immersion, the samples were rinsed with deionized water and dried in air at 70 °C (shown in Figure 1).

### 2.2. Characterizations

A Zeiss GeminiSEM500 field-emission scanning electron microscope (SEM, Carl Zeiss Microscopy GmbH, Jena, Germany) was used to observe the overall coverage status and morphology of the coatings, analyzing their surface uniformity and continuity. The elemental composition of the coatings was preliminarily inferred from EDS results. The surface of the coating was scanned using the SKPFM in the Bruker MultiMode 8 (Bruker Corporation, Billerica, MA, USA) scanning probe microscope to analyze the surface phase structure and the corresponding Volta potential distribution. The Volta potential distribution map was analyzed using NanoScope Analysis (version 1.7) software. A pull-off adhesion tester (PosiTest AT-M, DeFelsko, Ogdensburg, NY, USA) was used. Polyurethane coatings were applied via spin coating onto both the 55AlZnMg substrate and the ZrCC surface, with a coating thickness of 50 μm (Measure using the German QNIX-4500 thickness gauge (Automation Dr. Nix GmbH & Co. KG, Cologne, Germany)). Pull-off force tests were conducted, with three parallel tests performed for each sample group.

## 3. Results and Discussion

### 3.1. Composition Characteristics of the Multi-Alloy Phase on the Surface of 55AlZnMg Coatings

**Figure 2:**
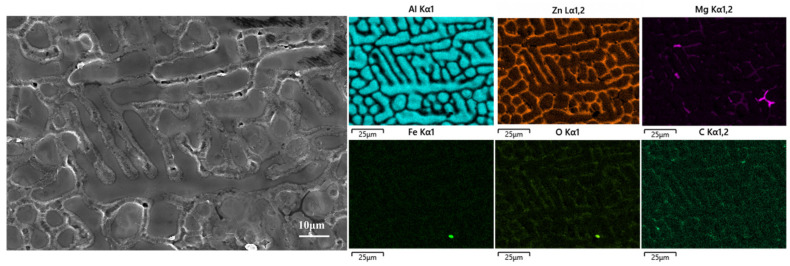
Distribution characteristics of each phase on the surface of 55AlZnMg coating: (×1000).

SEM-EDS analysis was used to examine the morphology and elemental distribution of the 55AlZnMg coating (Figure 2). Based on the elemental contrast and previous reports, the surface was assigned to Al-rich, Zn-rich, and Mg/Zn-enriched regions. Since no XRD analysis was performed, these regions are discussed as SEM-EDS-based compositional regions rather than crystallographically confirmed phases. Micrographs demonstrate that the Al-rich phase forms a continuous skeletal framework with a dendritic morphology. These dendrites, which nucleate first during solidification, constitute the primary structural support of the coating. The Zn-rich phases, containing minor amounts of MgZn_2_, are distributed within the interdendritic regions [12].

### 3.2. Potential Characteristics of the Multi-Alloy Phase on the Surface of 55AlZnMg Coatings

Owing to the complex multi-phase composition of the coating surface, the local electrochemical potentials dictate the selective deposition of film-forming species. To evaluate the deposition kinetics, the equilibrium electrode potentials for each phase in an H_2_ZrF_6_ solution (pH = 4) were calculated using the Nernst equation. These calculations identify the anodic and cathodic activities of the respective phases, providing a theoretical basis for investigating the preferential deposition sequence and kinetic mechanisms on the three distinct phases of the 55Al-Zn-Mg coating.

Taking the potential calculation for the aluminum phase (α-Al) as an example, α-Al undergoes the electrochemical reaction described in Equation (1a). Given the standard electrode potential E°(Al^3+^/Al) = −1.676 V(vs.SHE), the potential within the treatment solution is calculated using the Nernst equation (Equation (1b)), where *n* = 3. For a treatment solution at pH = 4, the proton concentration is [H^+^] = 10^−4^ M and the hydroxide concentration is [OH^−^] = 10^−10^ M. Assuming the Al^3+^ activity is governed by solubility equilibrium according to the dissolution of Al(OH)_3_ → Al^3+^ + 3OH^−^ (K_sp_ ≈ 10^−33^), the aluminum ion concentration is derived as [Al^3+^] = 10^−3^ M. Consequently, the calculated electrode potential for aluminum is E_Al_ = −1.499 V(vs.SHE).

2Al + 6H_2_O → 2Al(OH)_3_ + 3H_2_ (1a)

(1b) $$E=E{}^{\circ}({Al}^{3+}/Al)+\frac{0.05916}{n}\log\left(\frac{{Al}^{3+}}{{\left(H^{+}\right)}^{3}}\right)$$

Similarly, the electrochemical reaction occurring at the η-Zn phase is described by Equation (1c). Given the standard electrode potential E°(Zn^2+^/Zn) = −0.7618 V(vs.SHE), the potential within the treatment solution is calculated using the Nernst equation (Equation (1d)), where *n* = 2. The resulting electrode potential for zinc is determined to be E_Zn_ = −0.703 V(vs.SHE).

Zn + 2H^+^ → Zn^2+^ + 2H_2_ (1c)

(1d) $$E=E{}^{\circ}({Zn}^{2+}/Zn)+\frac{0.05916}{n}log(\frac{{Zn}^{2+}}{{\left(H^{+}\right)}^{2}})$$

MgZn_2_ is an intermetallic compound whose electrode potential lies between that of pure Mg (E° = −2.372 V(vs.SHE)) and Zn. Alexander et al. [34] employed electrochemical techniques to determine the electrode potential of MgZn_2_ at pH = 4, reporting a value of −1.00 V(vs.SHE). Based on the aforementioned analysis, the relative potential ranking of the constituent phases and the resulting galvanic coupling effects can be delineated. The established order of electrode potentials is: η-Zn > MgZn_2_ > α-Al (Table 1).

### 3.3. Time-Dependent ZrCC Deposition and Growth on 55AlZnMg Coatings

**Figure 3:**
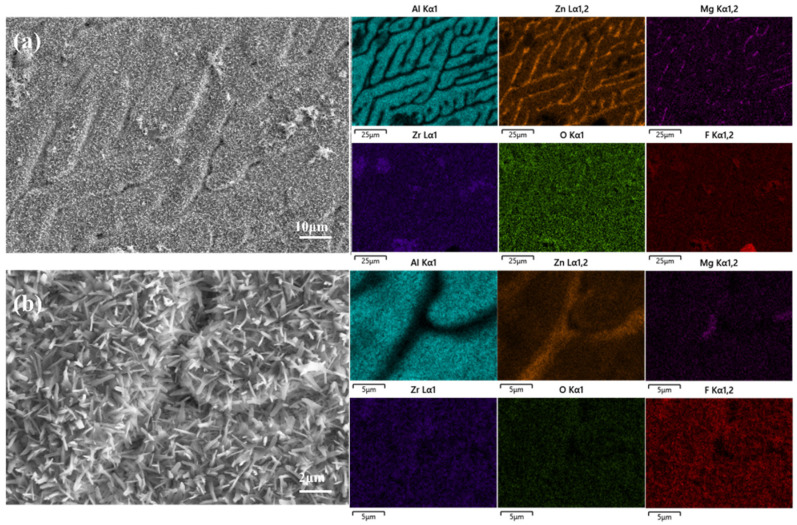
Microscopic morphology of the film layer and EDS scanning results after 20 s of film formation: (**a**) ×1000; (**b**) ×5000.

**Figure 4:**
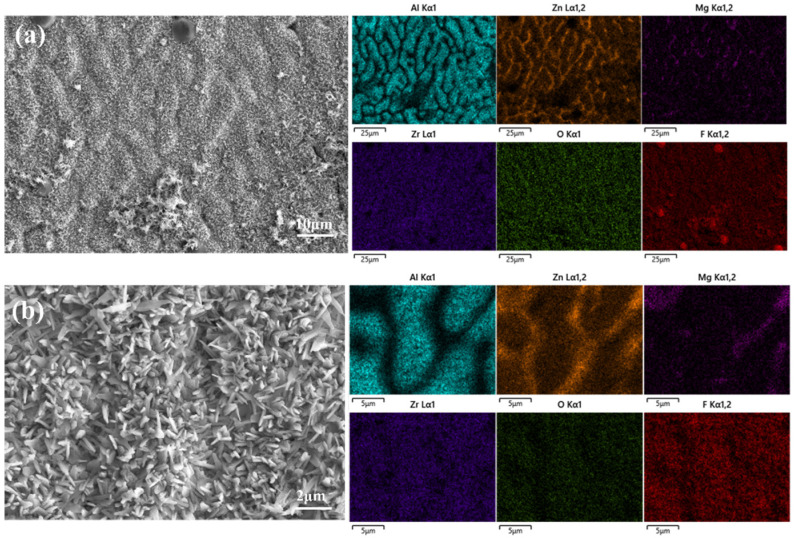
Microscopic morphology of the film layer and EDS scanning results after 30 s of film formation: (**a**) ×1000; (**b**) ×5000.

**Figure 5:**
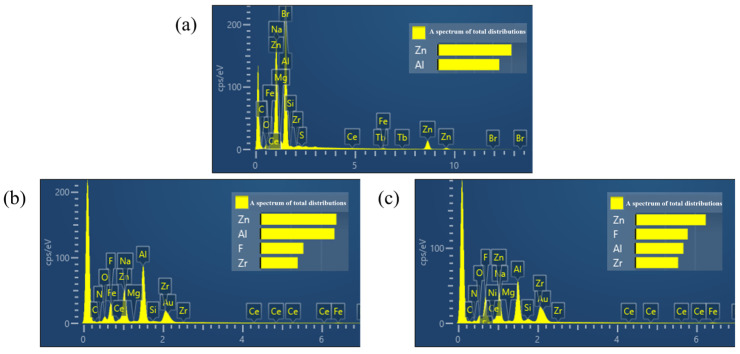
EDS spectra and semi-quantitative elemental contents of samples at different immersion times: (**a**) substrate; (**b**) 20 s; (**c**) 30 s. The elemental contents are given in wt.%.

**Figure 6:**
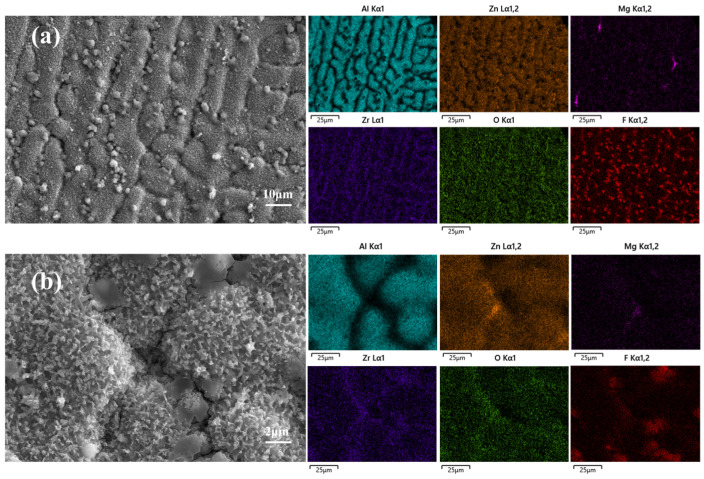
Microscopic morphology of the film layer and EDS scanning results after 40 s of film formation: (**a**) ×1000; (**b**) ×5000.

**Figure 7:**
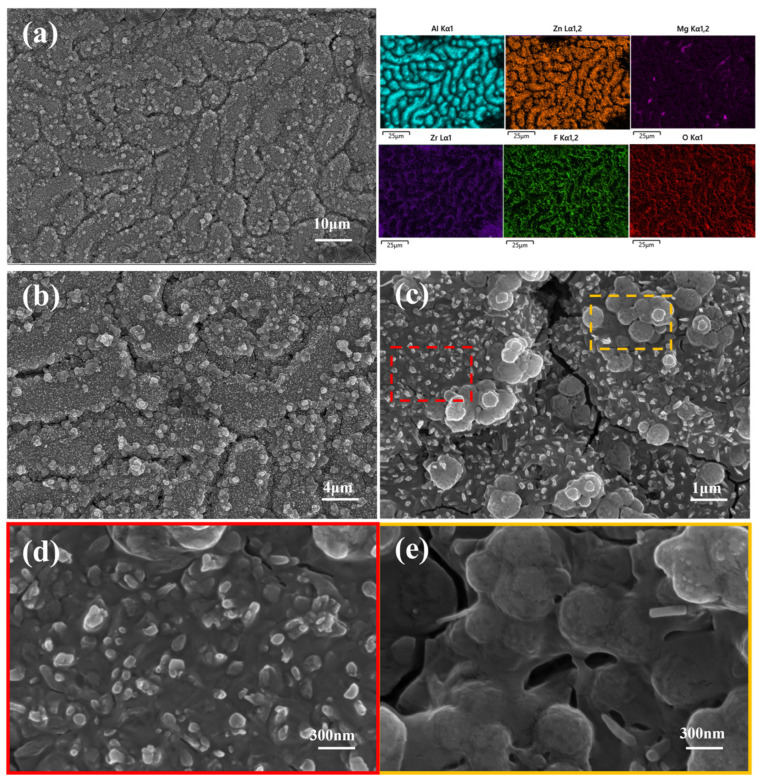
Microscopic morphology of the film layer after 60 s of film formation: (**a**) ×1000 morphology and EDS scanning results; (**b**) ×2500; (**c**) ×10,000; (**d,e**) ×30,000.

**Figure 8:**
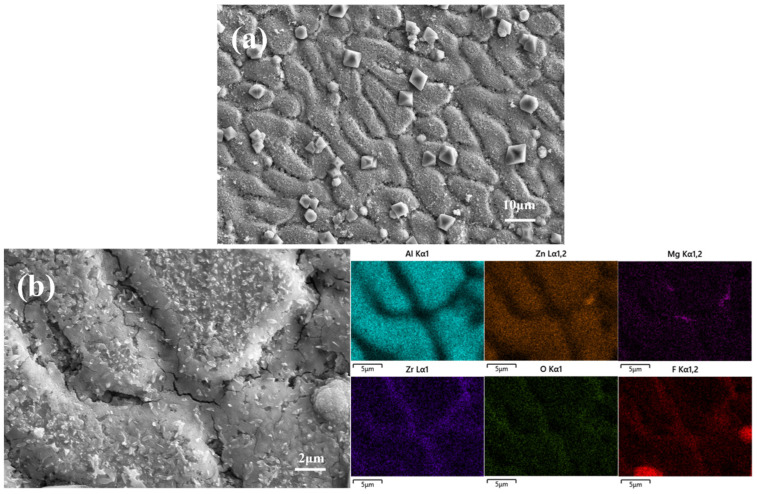
Microscopic morphology of the film layer after 90 s of film formation: (**a**) ×1000; (**b**) ×5000 Mor-phology and EDS scanning results.

To investigate the early-stage deposition kinetics of zirconium-based conversion coatings (ZrCC) on the 55AlZnMg substrate, panels were immersed in an additive-free 4% H_2_ZrF_6_ solution for durations between 20 and 90 s. The corresponding microstructural evolution and elemental distributions at these intervals are illustrated in Figure 3, Figure 4, Figure 5, Figure 6, Figure 7 and Figure 8.

Surface morphology and EDS mapping after 20 s of immersion (Figure 3) reveal micrometer-scale agglomerates of fluorine-containing compounds on the substrate, signifying the initiation of conversion coating deposition. This process is attributed to the aggressive attack of F^−^, which dissolves the native oxide film on the 55AlZnMg surface. The subsequent exposure of Zn-rich and Al-rich phases creates local galvanic micro-cells driven by the potential difference between these phases, thereby promoting the rapid nucleation of the conversion coating.

Micro-scale elemental analysis of the Zr distribution reveals a distinct depletion of Zn within Zr-enriched regions. Moreover, as illustrated in Figure 5a,b, the surface Zn/Al atomic ratio after 20 s of immersion is significantly lower than that of the pristine substrate. This indicates that Zr species preferentially deposit on the more noble (cathodic) Zn-rich phases. The co-localization of Zr and O suggests the formation of Zr/O-containing conversion products, the resulting coverage by a thin layer effectively masks the underlying Zn, thereby reducing its detected surface concentration.

Following 30 s of immersion (Figure 4), the film structure remains dominated by acicular compounds. Compared to the 20 s interval, surface coverage increases significantly as the deposition reaction progresses. SEM micrographs at 5000× magnification reveal that the conversion coating preferentially populates regions of higher microscopic electrochemical activity, resulting in a marked reduction in surface roughness. This observation indicates that the coating is undergoing a phase of accelerated deposition and densification.

Furthermore, a comparison of Figure 5b,c shows that surface Al content decreases significantly at 30 s relative to the 20 s mark, while the proportions of F and Zr increase (detailed results can be found in Table 2). This suggests that the deposition front of Zr species has propagated from the active Zn-rich phases toward the chemically more stable Al-rich matrix. Concurrently, the increased apparent deposition amount of the Zr and fluoride layers, alongside the growth of fluorine-containing agglomerates, facilitates the formation of a more compact surface layer.

Following 40 s of immersion (Figure 6), the microstructure of the conversion coating undergoes a significant transition, evolving from an initially loose acicular morphology into honeycomb-like porous nano-aggregates with a developed micro/nanostructured morphology. Simultaneously, dense, dark-gray spherical products are observed precipitating within the dendritic interstices of the coating. EDS elemental mapping reveals that these micrometer-scale spherical deposits, which form at the interface between Zn-rich and Al-rich phases, are fluorine-containing compounds that have evolved from the clustered aggregates observed at the 20 s and 30 s intervals.

EDS analysis further indicates the formation of extensive zinc-enriched regions, with surface area coverage exceeding that of the Al-rich phase in the substrate. This phenomenon is attributed to the unique phase composition of the 55AlZnMg coating. The proposed mechanism is as follows: Al within the Al-rich phase undergoes preferential anodic dissolution (i.e., the dealloying effect), exposing Zn within these micro-regions. This “in situ generated” Zn-enriched layer significantly enhances local cathodic activity, which in turn accelerates the generation of OH^−^ at the coating/solution interface. This promotes the transformation of Zr species from Zr-F complexes into OH^−^ bound compounds. This distinctive “dealloying-induced deposition” mechanism substantially facilitates the formation of zirconium-based conversion coatings on 55AlZnMg steel, enabling rapid expansion of the coating coverage. As illustrated in Figure 6, Zr is localized within the interstitial sites between the Zn and Al phases, existing predominantly as Zr/O-containing conversion products that occupy the grain boundary network.

Upon extending the immersion time to 60 s, the substrate SEM results (Figure 7) reveal a morphological transition toward a nanosheet-like structure. Two distinct particle types are evident on the surface: micron-sized spherical particles and nano-sized irregular white particles. The micron-sized particles are primarily distributed at the interface between the Zn-rich and Al-rich phases; based on the analysis in Figure 6, these are identified as fluoride precipitates. Notably, these particles are no longer merely superficially deposited but have become embedded within the coating. This transition indicates that the conversion coating has progressed beyond the initial nucleation stage into a phase of structural densification and refinement.

The nano-sized white particles dispersed within the Al-rich phase are attributed to the deposition of Zr-containing compounds. Their formation stems from the dealloying of the Al-rich phase in the 55AlZnMg coating, which induces secondary Zn enrichment in local micro-regions. This enrichment significantly enhances local cathodic activity, driving the redeposition of Zr-containing compounds at the original Al-rich sites. This “dealloying-redeposition” synergistic mechanism ultimately facilitates the rapid formation of a continuous zirconate conversion coating across the entire surface.

Figure 8 illustrates the SEM micrographs of the substrate after 90 s of immersion. At this stage, the morphology and interfacial bonding of the fluoride particles underwent significant changes, evolving from the initial spherical structures into larger, regular polygonal geometries. While the conversion coating appeared complete, a higher density of loosely adhered particles was observed on the surface, indicating weakened adhesion. Furthermore, despite the overall increase in coating density and deposition amount, localized micro-cracks emerged within the Zr^−^ and F-rich regions. This phenomenon is tentatively attributed to internal stresses generated within the film and at the coating–substrate interface during the formation of the zirconium-based layer. These stresses stem from film shrinkage, crystallization, and the coefficient of thermal expansion (CTE) mismatch between the coating and the substrate. Additionally, the increased thickness associated with prolonged immersion likely exacerbated volume contraction during the subsequent drying phase, further inducing stress-related localized cracking.

Comprehensive analysis of the micro-morphological evolution and elemental distribution demonstrates that, under the specific conversion bath conditions employed, zirconate coating deposition on the 55AlZnMg surface is effectively completed within 60 s. This result represents a significant reduction in the overall film formation time, highlighting the efficiency of the optimized process.

### 3.4. Role of the Selected H_2_ZrF_6_ Pretreatment Bath in Rapid Coating Formation

Under the selected H_2_ZrF_6_ pretreatment condition, a continuous ZrCC layer was formed on the 55AlZnMg surface within 60 s, as evidenced by the time dependent SEM-EDS observations. This result indicates that the present bath condition is effective for promoting short time conversion coating formation on the multiphase 55AlZnMg surface. However, because the H_2_ZrF_6_ concentration was not systematically varied in this study, the concentration effect should not be regarded as an independently verified controlling factor.

The rapid deposition observed under the present condition may be related to the availability of zirconium fluoride species in the treatment bath and the local alkalization induced by phase dependent micro-galvanic reactions on the 55AlZnMg surface. As the local interfacial pH increases near cathodically active regions, zirconium fluoride complexes may undergo hydrolysis and precipitation, leading to the formation of Zr/O-containing conversion products:

ZrF_6_^2−^ + 4OH^−^ → ZrO_2_·2H_2_O + 6F^−^ (1e)

This reaction provides a possible chemical basis for the deposition of zirconium-containing products. Nevertheless, in the absence of concentration dependent experiments conducted at constant pH, temperature, and immersion time, the present results only demonstrate that rapid ZrCC formation occurs under the selected H_2_ZrF_6_ bath condition. Further systematic studies are required to clarify the specific role of H_2_ZrF_6_ concentration in regulating deposition kinetics.

### 3.5. Influence of Alloy Phase Surface Characteristics on Conversion Coating Formation

To investigate the influence of alloy phases on the development of the conversion coating on the 55AlZnMg surface, atomic force microscopy (AFM) was utilized to characterize the morphology at various formation intervals. The fundamental film formation mechanism was elucidated by correlating the evolution of surface topography with the electrochemical potential variations between the constituent alloy phases within the 55AlZnMg coating.

**Figure 9:**
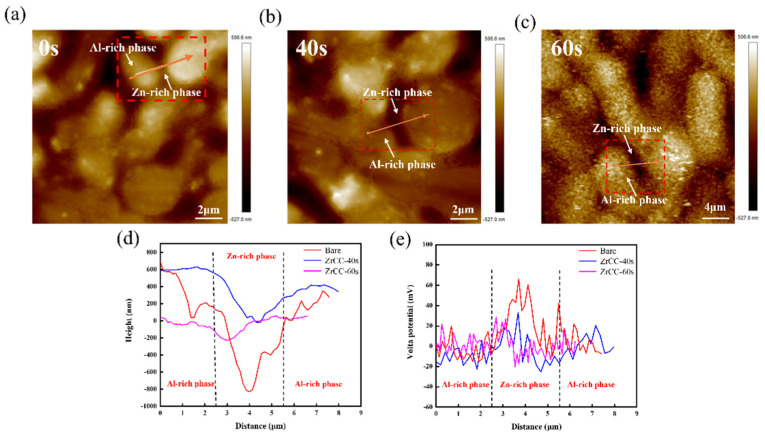
AFM test results of ZrCC: (**a**) 0 s; (**b**) 40 s; (**c**) 60 s; (**d**) Height distribution map; (**e**) Volta potential distribution map.

Conversion coatings were prepared on 55AlZnMg substrates at 40 s and 60 s intervals and characterized via AFM, utilizing the bare substrate as a control (Figure 9a–c, Table 3). Topographic analysis reveals that the Al-rich and Zn-rich phases remain clearly distinguishable across all samples, confirming that the nanometer-scale coating is too thin to effectively mask intrinsic phase-related height variations or surface defects. Notably, the 60 s sample displays a distinct “granular matte texture” compared to the 40 s sample. Correlation with the microstructural data in Figure 7d corroborates that, by 60 s, the surface is uniformly and densely encapsulated by a zirconate conversion coating composed of Zr-containing nanoparticles.

Comparison of the surface height and potential distribution curves reveals that, in its as-received state, the Zn-rich phase of the bare substrate exhibits a distinct “valley-like” morphology with a significant topographical recession relative to the Al-rich phase. This micro-feature is likely inherent to the solidification process of the alloy phases within the 55AlZnMg coating (Figure 9d). As immersion time increases, the topographical disparity between the two phases gradually diminishes. The decrease from approximately 600 nm at 40 s to 200 nm at 60 s signifies a marked enhancement in surface planarity.

Correlation with the micro-area potential analysis (Figure 9e) suggests that during the initial stages of film formation, the Zn-rich phase acts as the cathodic site (possessing a more noble potential). The resulting localized reduction reactions induce a sharp increase in micro-environmental pH, driving the hydrolysis and precipitation of zirconium-fluoride complexes. Consequently, conversion coating clusters nucleate and grow preferentially within these “valleys.” This “preferential filling effect” significantly increases coating deposition amount over the Zn-rich regions, effectively mitigating the original substrate’s topographical variations.

Furthermore, the temporal evolution of the surface potential indicates that the progressive coating coverage reduces the potential gradient between the Zn-rich and Al-rich phases. By 60 s, this potential difference is effectively neutralized, signifying the elimination of electrochemical heterogeneity. At this stage, the film formation process is largely complete, the chemical driving force subsides, and the growth phase concludes, consistent with the morphological observations in Figure 7.

### 3.6. Influence of Conversion Coatings on the Surface Characteristics of Plated Layers

Surface roughness and electrochemical homogeneity are fundamental determinants of the interfacial adhesion between a substrate and its coating. While moderate roughness facilitates mechanical interlocking, significant topographical disparities in multiphase alloys (such as the undulations between the Zn-rich and Al-rich phases in 55AlZnMg) can induce localized stress concentrations. These concentrations often trigger micro-cracking or delamination, ultimately compromising the bond strength. Moreover, electrochemical heterogeneity promotes micro-galvanic coupling, leading to localized corrosion or passive film instability, which further degrades the interfacial integrity [17,35].

To further elucidate the influence of the conversion coating on the interfacial characteristics of 55AlZnMg, polyurethane (PU) coatings were applied to both pristine and ZrCC-treated substrates. Pull-off testing revealed that the adhesion strength of the pristine substrate was 5.03 MPa; however, following ZrCC treatment, the interfacial bond strength significantly increased to 8.25 MPa, representing a 64.0% enhancement. This substantial improvement confirms the pivotal role of the conversion coating in establishing a robust adhesion promoting interface.

Given that the adhesion between the conversion coating and the PU layer is governed by both mechanical interlocking and chemical bonding, the observed increase in bond strength is attributed to the following factors: (1) Post-ZrCC treatment, the film preferentially deposits within the “valleys” of the Zn-rich phase, significantly mitigating the initial interphase topographical disparity of the 55AlZnMg substrate. This process constructs a “blunted-peak/shallow-valley” micro-nano-structure composed of Zr-containing nanoparticles. These features serve as effective mechanical anchors while simultaneously eliminating stress concentrations typically induced by macroscopic interphase undulations. This facilitates a uniform interfacial stress distribution, thereby significantly enhancing the mechanical bond between the coating and the substrate [24]. (2) The presence of Zr/O-containing conversion products increases surface polarity compared to the untreated substrate [24]. The treated surface possesses a high density of active functional groups (primarily adsorbed fluoride anions and hydroxyl groups [36]) which significantly elevates the surface energy. This polar surface facilitates the formation of robust hydrogen and covalent bonds with the reactive functional groups of the PU coating, thereby strengthening interfacial chemical interactions [37]. (3) In the pristine 55AlZnMg substrate, the significant potential difference between the Al-rich and Zn-rich phases predisposes the surface to micro-galvanic coupling. Following ZrCC treatment, however, the surface potential is effectively homogenized, eliminating distinct anodic and cathodic regions. This suppression of micro-galvanic activity may reduce electrochemical heterogeneity at the interface, resulting in a stable interface less susceptible to localized delamination. Consequently, the average adhesion and the interfacial stability is expected to be improved.

In conclusion, the Zr-based conversion coating significantly enhances the interfacial adhesion between the organic coating and the 55AlZnMg-coated steel substrate via a tripartite synergistic mechanism comprising topographical reconstruction for mechanical interlocking, chemical bonding enhancement, and electrochemical potential homogenization. This synergistic effect establishes a robust foundation for the subsequent deposition of composite coatings, providing a favorable interface for subsequent organic coating deposition.

### 3.7. Deposition Kinetics of Conversion Coatings

Based on the analysis of alloy phase potentials and AFM results for the 55AlZnMg-coated steel, and grounded in electrochemical interphase coupling theory, the deposition mechanism of the conversion coating is proposed as follows: The conversion bath contains active F^−^ that etches the native oxide layer, exposing the underlying Zn-rich and Al-rich phases. Within this micro-galvanic couple, α-Al serves as the anode and undergoes preferential dissolution, releasing electrons that migrate to the η-Zn cathodic sites. On the η-Zn surface, hydrogen evolution and oxygen reduction reactions occur sequentially; while hydrogen evolution predominates in the initial acidic environment, oxygen reduction becomes dominant as the local pH rises. These cathodic reactions induce localized surface alkalinization, triggering the selective precipitation of film-forming species such as Zr/O-containing conversion products. Concurrently, the high electrochemical activity of Al and Mg within the α-Al phase and MgZn_2_ intermetallic compounds promotes preferential anodic dissolution, leading to selective dealloying. The continuous leaching of Al^3+^ and Mg^2+^ results in dynamic Zn enrichment at the phase interfaces. This dealloying process exerts an autocatalytic effect via a micro-galvanic mechanism: the newly formed Zn-enriched regions function as secondary cathodes, further accelerating anodic dissolution in adjacent areas. The local alkalization caused by cathodic reactions is expected to promote the hydrolysis of zirconium-fluoride complexes and the precipitation of zirconium-containing products, promoting rapid coating deposition. Ultimately, this self-sustaining electrochemical process drives the coating’s expansion from localized nucleation to complete surface coverage. By regulating the potential in Zn-enriched regions, yielding a continuous and relatively uniform conversion layer across the 55AlZnMg surface.

**Figure 10:**
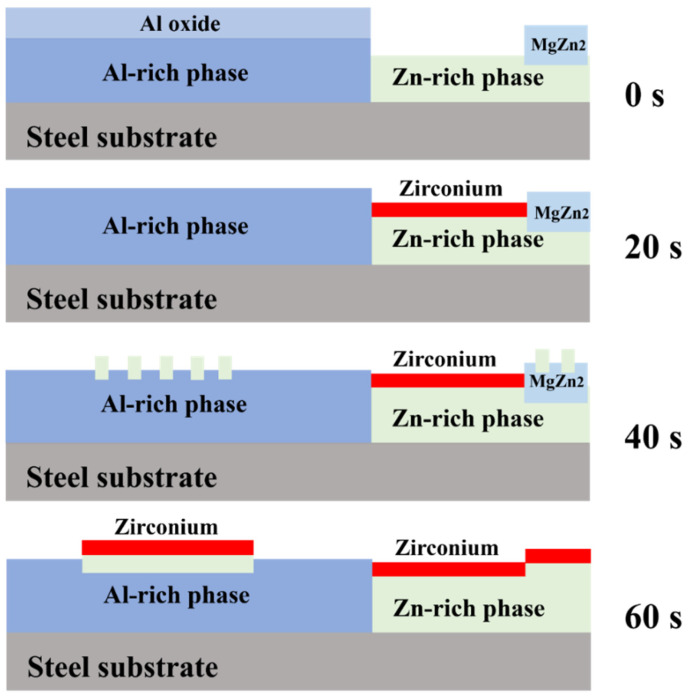
Diagram of the film formation mechanism of ZrCC on 55AlZnMg.

Figure 10 illustrates the proposed mechanism by correlating the morphological observations with the film formation stages. The phase-dependent deposition of ZrCC on the 55AlZnMg surface is closely related to the local electrochemical heterogeneity of the Al-rich, Zn-rich, and Mg/Zn enriched regions. The relatively noble Zn-rich regions may act as preferential cathodic sites, where hydrogen evolution and oxygen reduction can locally increase the interfacial pH:

2H^+^ (aq) + 2e^−^ ⇄ H_2_ (g) (1f)

O_2_ (g) + 2H_2_O + 4e^−^ ⇄ 4OH^−^ (aq) (1g)

Concurrently, the localized OH^−^ enrichment facilitates the hydrolysis of ZrF_6_^2−^ complex ions, inducing the deposition of zirconium-based species while destabilizing the Zr-F coordination [29].

[ZrF_6_]^2−^ + 2H_2_O → ZrO_2_ (s) + 4H^+^ + 6F^−^ (1h)

The hydrolyzed Zr-containing species may subsequently contribute to the formation of Zr/O-containing conversion products on the substrate surface. Meanwhile, anodic dissolution of the Al-rich and Mg/Zn enriched regions may occur during the dealloying process. Consequently, Zn particles accumulate on the surface, establishing Zn-enriched regions (represented by the light green particles in the figure). These Zn-enriched domains function as secondary cathodic sites that facilitate the previously described hydrogen evolution and oxygen reduction reactions, resulting in localized surface alkalinization that promotes the precipitation of film-forming species. By 60 s, the conversion coating achieves near-complete surface coverage.

Al − 3e^−^ ⇄ Al^3+^ (1i)

Mg − 2e^−^ ⇄ Mg^2+^ (1j)

The released F^−^ may further participate in possible solution complexation reactions with dissolved metal cations:

Zn^2+^ + 4F^−^ ⇄ [ZnF_4_]^2−^ (1k)

Al^3+^ + 6F^−^ ⇄ [AlF_6_]^3−^ (1l)

These reactions should be regarded as possible interfacial and solution-chemistry processes involved in ZrCC formation.

## 4. Conclusions

This study investigated the deposition kinetics of zirconium-based conversion coatings (ZrCC) on 55AlZnMg surfaces by correlating theoretical alloy phase potential calculations with SEM-EDS and AFM characterizations. By synthesizing micro-morphological observations with these electrochemical data, the early-stage film formation mechanism of the ZrCC/55AlZnMg system was elucidated, providing a mechanistic basis for short-time ZrCC pretreatment on 55AlZnMg.

1. With increasing immersion duration, the surface morphology transitions from acicular structures to honeycomb-like porous nano-aggregates, characterized by enhanced structural compactness. After 60 s of immersion, the coating develops a nanosheet-like morphology and achieves its maximum density. Conversely, extending the immersion time to 90 s induces localized micro-cracks, leading to a subsequent degradation in coating integrity.

2. The multiphase surface characteristics of the 55AlZnMg coating play a decisive role in the rapid and relatively uniform formation of the ZrCC layer. Based on SEM-EDS observations, the surface can be assigned to Al-rich, Zn-rich, and Mg/Zn-enriched regions, which exhibit different electrochemical activities during immersion in the H_2_ZrF_6_ bath. The Zn-rich regions, with relatively noble potential and recessed topography, act as preferential sites for the initial deposition of Zr/O- and F-containing conversion products. Meanwhile, the selective dissolution and dealloying of Al-rich and Mg/Zn-enriched regions promote local Zn enrichment, generating secondary cathodic sites that sustain interfacial alkalization and further deposition. Therefore, the substrate multiphase structure is not an isolated compositional feature, but the key factor governing the phase-selective growth and rapid surface coverage of ZrCC on 55AlZnMg.

3. The ZrCC treatment effectively modified the surface characteristics of the 55AlZnMg coating, mitigating topographical irregularities and electrochemical heterogeneity via a tripartite synergistic mechanism involving mechanical interlocking, enhanced chemical bonding, and potential homogenization. Following ZrCC treatment, the interfacial adhesion between the 55AlZnMg substrate and the polyurethane coating increased from 5.03 MPa to 8.25 MPa, representing a 64.0% enhancement in adhesion.

In summary, the rapid 60 s ZrCC process developed in this study effectively addresses the inherent non-uniformity of film formation on multiphase Al-Zn-Mg coatings. These findings establish a comprehensive theoretical and technical framework for optimizing surface uniformity and interfacial adhesion in short-time ZrCC pretreatment processes.

## Figures and Tables

**Table 1:** Electrode potentials of each phase of 55AlZnMg alloy.

Phase	Potential/V	Description
α-Al	−1.499	Anode (priority dissolution)
MgZn_2_	−1.000	Secondary anode
η-Zn	−0.703	Cathode (electron acceptor)

**Table 2:** Semi-quantitative EDS results of the substrate and ZrCC-treated samples.

	Zn/wt.%	Al/wt.%	F/wt.%	Zr/wt.%	Other/wt.%
Substrate	53.8	44.3	-	-	1.9
20 s	33.2	31.8	18.6	15.8	0.6
30 s	32.8	22.1	23.8	19.8	1.5

**Table 3:** The roughness parameters were calculated from the leveled SKPFM height profiles. R_a_ is the arithmetic average roughness, R_q_ is the root mean square roughness, R_z_ is the maximum peak-to-valley height, R_sk_ is the skewness, and R_ku_ is the kurtosis.

Sample	R_a_/nm	R_q_/nm	R_z_/nm	Skewness, R_sk_	Kurtosis, R_ku_
0 s	308.0	373.9	1346.3	−0.665	2.464
40 s	144.8	171.7	565.1	−0.949	2.522
60 s	58.7	75.5	308.3	−1.090	3.259

## Data Availability

The original contributions presented in this study are included in this article. Further inquiries can be directed to the corresponding author.
